# Sericin/crocetin micro/nanoparticles for nucleus pulposus cells regeneration: An “active” drug delivery system

**DOI:** 10.3389/fphar.2023.1129882

**Published:** 2023-03-10

**Authors:** Elia Bari, Sara Perteghella, Giovanna Rassu, Elisabetta Gavini, Giacomo Luigi Petretto, Maria Cristina Bonferoni, Paolo Giunchedi, Maria Luisa Torre

**Affiliations:** ^1^ Department of Pharmaceutical Sciences, University of Piemonte Orientale, Novara, Italy; ^2^ Department of Drug Sciences, University of Pavia, Pavia, Italy; ^3^ Department of Medicine, Surgery and Pharmacy, University of Sassari, Sassari, Italy

**Keywords:** silk-sericin, crocetin, nucleus pulposus regeneration, nanoparticles, oxidative stress damage, antioxidant

## Abstract

**Introduction:** Initiation and progression of intervertebral disk degeneration are linked to oxidative stress, with reactive oxygen species being a key factor. Therefore, as a potentially novel approach able to regenerate the damaged intervertebral disk, this work aimed to prepare an “active per sé” drug delivery system by combining sericin and crocetin: both are bioactive compounds with antioxidant, anti-inflammatory, immunomodulant and regenerative properties.

**Methods:** In detail, sericin nanoparticles were prepared using crocetin as a cross-linker; then, the nanoparticle dispersions were dried by spray drying as it is (NP), with an excess of sericin (NPS) or crocin/crocetin (NPMix), obtaining three microparticle formulations.

**Results and Discussion:** Before drying, the nanoparticles were nanometric (about 250 nm), with a negative surface charge, and appeared spherical and smooth. Following the drying process, spherical and smooth microparticles were obtained, with a mean diameter of about 1.7–2.30 μm. NPMix was the most active in antioxidant and anti-tyrosinase activities, likely due to the excess of crocin/crocetin, while NPS had the best anti-elastase activity, likely due to sericin in excess. Furthermore, all the formulations could prevent oxidative stress damage on nucleus pulposus cells, with NPMix being the best. Overall, the intrinsic anti-tyrosinase and anti-elastase activities and the ability to protect from oxidative stress-induced damages justify future investigations of these “active per sé” formulations in treating or preventing intervertebral disk degeneration.

## 1 Introduction

The intervertebral disk is a fibrocartilaginous structure that acts as a joint between the adjacent vertebral bodies, to which it is connected by cartilage plates (Chan et al., 2011). It is characterized by a peripheral part with a lamellar structure, the annulus fibrous, and a central part, the nucleus pulposus, and its primary functions are to assure the spine’s articularity and to absorb the shocks and stresses to which the column is inevitably subjected daily, such as compressions with torsional, flexural and extensional components (Lundon and Bolton, 2001).

Following ageing and other environmental factors, such as abnormal mechanical stress, the cellular components of the intervertebral disk undergo substantial biological changes, such as cell de-differentiation, with consequent loss of the characteristic phenotype and a decrease in the number of viable cells (De Geer, 2018b). All of this ultimately leads to an impairment in the synthesis of the components of the matrix, especially proteoglycans (Roughley, 2004; Wei et al., 2019) and an increase in catabolic metabolism, mainly operated by metalloproteases and cathepsins (Colombini et al., 2008; Liang et al., 2022). Also, a change in the secretion of growth factors (Sun et al., 2020) and increased pro-inflammatory cytokines generally occur (De Geer, 2018a). Therefore, intervertebral disk degeneration is not a passive wear process but an aberrant and cell-mediated response that causes progressive structural failure with ultimate rupture of the extracellular matrix; according to the more recent findings, the initiation and progression of all these processes are linked to oxidative stress (Xiang et al., 2022). Especially in recent years, numerous studies have demonstrated that reactive oxygen species (ROS) were highly expressed in intervertebral disks in animal or human degenerative samples, and were key factor for the initiation and development of intervertebral disk degeneration (Nasto et al., 2013; Hou et al., 2014; Davalli et al., 2016).

The current therapy for intervertebral disk degeneration involves conservative measures and surgery. The first approach requires drug therapy with non-steroidal anti-inflammatory drugs and muscle relaxants associated with a physiokinesitherapy program to improve the range of motion and postural control (Romaniyanto et al., 2022). The surgical treatment is provided only in the event of failure of conservative therapy with the persistence of symptoms for at least 3 months, in the case of worsening neurological deficits or in the case of cauda equina syndrome (Dou et al., 2021). More recently, potential novel approaches focused on regenerating the damaged intervertebral disk by preventing cell differentiation, increasing cell proliferation and counteracting oxidative stress damage. As recently reviewed, these effects may be achieved using growth factors, small molecules, gene therapy, and stem cells (Romaniyanto et al., 2022). For example, in both *in vitro* and animal intervertebral disk tissue engineering studies, some growth factors, such as IGF-1, GDF-5, BMP-2, BMP-7 and platelet-derived ones, have shown the ability to modulate the anabolic and anticatabolic effects (Kennon et al., 2018). In this regard, in a previous paper, we proposed the combined use of sericin microparticles and platelet lysate, a blood-derived product rich in growth factors, as a therapeutic strategy to support the regeneration of the intervertebral disk. Sericin was selected as a carrier due to its low toxicity, biocompatibility, and biodegradability but also biological properties, including antioxidant, anti-inflammatory, immunomodulant and regenerative effects (Bari et al., 2018).

Sericin is an adhesive protein, a component of silk produced by various species of insects, such as arachnids, scorpions and bees, and especially by the silkworm *Bombyx mori* (Bari et al., 2020). Due to its favourable properties, sericin is achieving greater attention in regenerative medicine, and it is widely studied for the formulation of drug delivery systems, like hydrogels, microparticles, and nanoparticles. However, the possibility of using only pure sericin to generate scaffolds and drug delivery systems is often hampered by its high solubility in water. Therefore, sericin usually has to be copolymerized, cross-linked, or blended with other polymers to increase its stability. In this regard, we recently proposed to cross-link sericin with crocetin (Perteghella et al., 2021), a carotenoid derived from the hydrolysis of crocin, a molecule present in saffron and in the fruits of *Gardenia jasminoides Ellis* (Hashemi and Hosseinzadeh, 2019). Crocetin is a non-toxic substance that additionally has different therapeutic properties, like antitumor (prevents cell proliferation and increases apoptosis in cancer cells) (Colapietro et al., 2019), neuroprotective action (Scuto et al., 2022) and activity on the cardiovascular system, for example, in the treatment and prevention of arterial hypertension, thrombosis and myocardial infarction (Kadoglou et al., 2021). These benefits are mainly attributed to the antioxidant properties of that molecule (Cerda-Bernad et al., 2022).

Given these premises, we supposed that thanks to their biological properties, sericin and crocetin could act synergistically in the regenerative processes of the degenerated intervertebral disk. Therefore, this work aimed to formulate, prepare, and characterize sericin and crocetin nanoparticles that were then spray-dried to obtain microparticles. Three different formulations were prepared, spray drying the only nanoparticles (NP) and the nanoparticles with an excess of sericin (NPS) or crocetin (NPMix). All the formulations have been tested *in vitro* to evaluate their antioxidant, anti-tyrosinase and anti-elastase activity, as well as the ability to protect the cells of the nucleus pulposus from damage induced by oxidative stress.

## 2 Materials and methods

### 2.1 Materials

2, 2-Diphenyl-1-picrylhydrazyl (DPPH), 2-propanol, 3-(4,5-Dimethylthiazol-2-yl)-2,5-Diphenyltetrazolium Bromide (MTT), arbutin, crocin, epigallocatechin gallate (E), N-Succinyl-Ala-Ala-Ala-p-Nitroanilide, porcine pancreatic elastase (PPE) and tyrosinase, were purchased from Merck, Milan, Italy. Ethanol, methanol, dimethyl sulfoxide (DMSO), hydrogen peroxide (H_2_O_2_) and sodium hydroxide (NaOH) were purchased from Carlo Erba Reagents, Milan, Italy. Hydrochloric acid (HCl) and ferric sulphate (Fe_2_(SO_4_)_3_) were bought from VWR chemicals, Milan, Italy. All the cell culture reagents were purchased from Euroclone, Milan, Italy.

### 2.2 Sericin extraction and purification

*Bombyx mori* cocoons were divided into 1 × 1 cm pieces and put through an autoclave degumming procedure (Systec V-65, Wurttemberg, Germany) for 1 h at 120°C (40 mL of water for each g of cocoons). The sericin solution was then filtered through 70 μm cell sieves (Thermo Fisher Scientific, Milan, Italy) to remove significant contaminants (e.g., fibroin fibres), frozen at −80°C, and freeze-dried for 72 h (Modulyo^®^ Edwards Freeze drier, Kingston, New York, NY, United States). The resulting powder was kept at −20°C for storage. Before usage, sericin powder was dissolved in MilliQ water (5 mg/mL) and heated to between 70°C and 80°C while constantly stirring with a magnetic device for 2 h. Next, the mixture was cooled and centrifuged (Eppendorf Centrifuge 5702 R, Eppendorf, Germania) for 10 min at 4400 rpm at 4°C. The operation was repeated three times to eliminate all the impurities.

### 2.3 Preparation of sericin nanoparticles using crocetin as a cross-linker

As detailed below, three formulations were prepared (NPMix, NPS and NP). The reproducibility of the formulations was established by preparing three batches for each.

#### 2.3.1 Preparation of NPMix

Crocin (240 mg) was added to 10 mL of the 5 mg/mL sericin solution and solubilized in the dark by magnetic stirring. Then, 20 mL of ethanol was introduced by dripping under constant magnetic stirring to favour the protein’s desolvation. In the end, the suspension was alkalized drop by drop with NaOH 0.1 M up to pH eight to nine and placed in a 50°C water bath for 30 min to favour the hydrolysis of crocin to crocetin, and allow the last component to cross-link with sericin; at this stage, NPs are formed. Finally, the suspension (NPMix), containing the nanoparticles and free crocin and crocetin, was dried by spray drying to obtain a stable powder (see Section 2.4).

#### 2.3.2 Preparation of NP

Following the procedure described above, after hydrolysis, the nanoparticles were isolated. The suspension was centrifuged for 15 min at 4400 rpm at 4°C; after decanting, a brown-coloured and gelatinous pellet was obtained, and it was resuspended with MilliQ water with a ratio of 1:2 to the initial volume of sericin solution. An ultrasound probe (Sonics and Materials Inc., Danbury, CT, United States) was used for 30–40 min at 40% amplitude to favour the dispersion. Finally, the dispersed nanoparticles (NPs) were spray-dried using the conditions reported in Section 2.4.

#### 2.3.3 Preparation of NPS

The same procedure, including desolvation with ethanol, basic hydrolysis and isolation by centrifugation, was followed for preparing nanoparticles containing an excess of sericin (NPS). However, in this case, the obtained pellets were resuspended with a 10 mg/mL sericin solution with a ratio of 1:2 to the initial volume of sericin solution. Finally, the dispersion was dried by spray drying (see Section 2.4)

### 2.4 Drying of nanoparticles by spray drying

The dispersed nanoparticles were dried by spray drying (BUCHI Mini Spray Dryer B-191, BUCHI Labortechnik AG CH-9230, Flawil, Swiss) to preserve stability over time. The instrument’s parameters have been set so that the temperature difference between the inlet air (120°C) and the outlet air (80°C) is stable and should not exceed 40°C. The optimal conditions to maintain this balance included a 94% aspirator, a 5% pump and a compressed air flow of 500 L/h.

### 2.5 Characterization of the nanoparticles

#### 2.5.1 Evaluation of nanoparticle yield

To quantify the percentage of cross-linked sericin in the nanoparticles, the various components of the decanted supernatant were separated, as previously reported (Perteghella et al., 2021). Briefly, the supernatant was transferred into a flask and evaporated with the Rotavapor (Büchi 011, Labortechnik AG, Flawil, Svizzera) at 70°C–80°C; it was then placed in a ventilated oven (M4000, Memmert GmbHS Schwabach, Germania) at 45°C for one night. As a result, a red vitreous solid was obtained and treated with 0.2 M HCl. The solubility in 0.2 M HCl of the various starting products is reported in Table 1. At the precipitate, separated by centrifugation at 4000 rpm at 4°C, MilliQ water was added, and the tube was placed in a 70°C–80°C water bath for 1 h, under stirring. In this way, the sericin passed into the solution was transferred into a flask and evaporated by Rotavapor. The sericin recovered in the supernatant was related to the amount of sericin used for nanoparticle preparation for the yield, as previously described (Perteghella et al., 2021). The yield calculation was performed only for the NP and NPS formulations, where it was possible to isolate the nanoparticles.

**TABLE 1:** Comparison between the 0.2 M HCl solubilities of the substances used in the preparation of nanoparticles.

Component	Solubility in HCl 0.2 M
Sericin	Insoluble
Crocin	Soluble
Crocetin	Insoluble

#### 2.5.2 Dimensional analysis and stability studies

To evaluate the average diameter and the dimensional distribution of the nanoparticles in the different formulations, the Nanosizer (N5 Submicron Particle Size Analyzer, Beckman Coulter Inc., Brea, California) was used. Analyses were performed before and after the spray drying. Before spray drying, the samples were diluted with MilliQ water (previously filtered through a 0.22 μm regenerated cellulose filter) and introduced into the instrument. As regards the preparation of post-drying samples, 1 mL of MilliQ was added to 2 mg of powder; the suspension was dispersed by ultrasound probe with 40% amplitude for 1 min. The results are expressed as the diameter (nm) and the polydispersity index (PI), with the relative standard deviations. The results are the average of three determinations for each batch (*n* = 9).

The dimensional analysis of post-drying samples was repeated after dissolving 1 mg of powder in 0.5 mL of complete culture media (with the composition detailed in Section 2.7.4). Analyses were performed using the Nanoparticle Tracking Analysis technique (NanoSight NS300 equipment, Malvern Panalytical, Grovewood Rd, United Kingdom). The stability was evaluated 24, 48 and 72 h after resuspension by measuring the size of samples stored at 4°C or 37°C.

#### 2.5.3 Morphological evaluation by transmission electron microscopy (TEM)

TEM was performed using a JEOL JEM 1200 EX instrument. The sericin nanoparticles of each formulation were observed by placing a drop of the sample suspension (concentration = 0.1 mg/mL) onto a 300 mesh nickel grid coated with carbon. Before the measurement, the samples were left to dry for 24 h at room temperature (25°C).

#### 2.5.4 Determination of the surface charge

The Zeta potential of dried nanoparticles suspended in Milli-Q water was measured with a Zetasizer Nano ZS (Malvern Instruments, Malvern, United Kingdom) at room temperature. The results are the mean of three analyses.

### 2.6 Characterization of the microparticles

#### 2.6.1 Yield of the spray drying process

At the end of the spray drying process, the powder was transferred to a desiccator overnight and weighed. The yield was calculated as follows:

$$Yield\;\left(\%\right)=\frac{mg\;of\;obtained\;powder}{\sum mg\;of\;initial\;substances}$$

For the calculation of the drying yield of NPMix, the starting products’ totality was considered, while for NP and NPS, only the initial total sericin was considered.

#### 2.6.2 Dimensional analysis

The Coulter Laser Diffraction (Coulter LS 100Q, Coulter Corporation, Miami, Florida, United States) was used for the dimensional analysis of the microparticles: 2 mg of powder were dispersed in 1 mL of 2-propanol and sonicated in an ultrasonic bath; the suspension was added to the inside of the cell containing 13 mL of 2-propanol, up to the concentration required by the instrument. The results are expressed as surface-volume equivalent diameter (dvs) and mode with relative standard deviation.

#### 2.6.3 SEM morphological analysis

Morphological analysis was performed using a scanning electron microscope (SEM, FEI Quanta 200, FEI, Hillsboro, OR, United States) on NP, NPS and NPMix powders. The powders were placed inside the stub and analyzed without carrying out the gold coating; the images were acquired operating in a high vacuum and low voltage (5 kV) condition, with a spot size of 2.5.

#### 2.6.4 Quantification of crocin and crocetin in NPMix

NPMix (5 mg) was dissolved in 1 mL of a solution of 0.1 M HCl. The resulting mixture was centrifuged (Eppendorf Centrifuge 5702 R, Eppendorf, Hamburg, Germany) at 4400 rpm for 10 min at 4°C and the supernatant containing the free crocin was immediately analyzed by UV-Vis spectrophotometer (Ultrospec 71 4300 pro UV–VIS spectrophotometer) at 440 nm. First, the precipitate was dissolved in 1 mL of NaOH 0.1 M. Then, 5 mg of Fe_2_(SO_4_)_3_ as a chemical coagulant and adsorbent were added to allow nanoparticle flocculation. The resulting mixture was centrifuged at 4400 rpm for 10 min and 4°C, and the supernatant containing the free crocetin solution was immediately analyzed at 410 nm. Quantification of total crocin and total crocetin was obtained by external standard calibration method; calibration curves were obtained by reading the absorbance of crescent concentration work solutions of crocin in HCl 0.1 M and crocetin in NaOH 0.1 M. Standard crocetin was obtained by hydrolytic cleavage of the crocin glycosidic bonds; the reaction was carried out according to a previous literature method, with slight modification (Reddy et al., 2020). Briefly, a solution of crocin (100 mg) in 2 mL of NaOH 0.1M (5-fold excess) was stirred in the dark at 40°C for 30 min. After the reaction time, HCl 0.1 M was added until a precipitate appeared (pH < 7). The precipitate formed was filtered and washed with water to give the crocetin sample as a red-brown solid (yield 60%).

### 2.7 *In Vitro* biological activity

#### 2.7.1 ROS-scavenging activity

The ROS-scavenging activity of each formulation was evaluated by the DPPH colourimetric assay. 30 μL of the sample were combined with 270 μL of a DPPH solution (0.0056% w/v in 70% v/v methanol) to create the reaction mixture. For each sample, the final concentration in the reaction mix was 5, 2.5 and 1.25 mg/mL. A reaction mix without the sample was considered a negative control. After incubation in the dark for 5 min at room temperature, the absorbance of samples and negative control was measured at 515 nm by a microplate reader (Synergy HT, BioTek, Swindon, United Kingdom). The ROS-scavenging activity percentage was calculated according to the following formula:

$$ROS-scavenging\;activity\;\left(\%\right)=\frac{A_{ctr}-A_{samp}}{A_{ctr}}\times 100$$

where A_ctr_ is the absorbance of the negative control and A_samp_ is the absorbance of the sample. Each experiment was performed in triplicate.

#### 2.7.2 Anti-elastase activity

A spectrophotometric *in vitro* method was used to examine the anti-elastase activity. Briefly, the samples were initially incubated for 20 min in the dark at room temperature with the enzyme PPE previously solubilized in phosphate buffer pH 6.8 at 0.5 IU/mL. The final concentration of each sample in the reaction mix was 5, 2.5 and 1.25 mg/mL. As a positive control, a reaction mix containing epigallocatechin gallate (at a final concentration of 7.2 mg/mL in deionized water) was employed, while a reaction mix without the sample was used as a negative control. Then the substrate N-Succinyl-Ala-Ala-Ala-p-Nitroanilide was solubilized in TRIS buffer at 0.41 mM and added to the reaction mix of samples and controls. The absorbance was measured at 410 nm by a microplate reader (Synergy HT, BioTek, Swindon, United Kingdom) for 60 min (one measurement each minute). The anti-elastase activity percentage was calculated using the following formula:

$$Anti-elastase\;activity\;\left(\%\right)=\frac{A_{ctr}-A_{samp}}{A_{ctr}}\times 100$$

where A_ctr_ is the absorbance of the negative control and A_samp_ is the absorbance of the sample. All the analyses were performed in triplicate.

#### 2.7.3 Anti-tyrosinase activity

The anti-tyrosinase activity was investigated by a spectrophotometric *in vitro* method. Briefly, the samples were initially incubated for 10 min in the dark at room temperature with the enzyme tyrosinase previously solubilized in phosphate buffer pH 6.8 at 500 IU/mL. The final concentration of each sample in the reaction mix was 5, 2.5 and 1.25 mg/mL. A reaction mix without the sample was used as a negative control, while a reaction mix with arbutin (at the final concentration of 2.5 mg/mL in deionized water) was considered the positive control. Then the substrate L-tyrosine was solubilized in phosphate buffer pH 6.8 at 0.3 mg/mL and added to the reaction mix of samples and controls. The absorbance was measured at 480 nm by a microplate reader (Synergy HT, BioTek, Swindon, United Kingdom) for 60 min (one measurement each minute). The anti-tyrosinase activity percentage was calculated using the following formula:

$$Anti-tirosinase\;activity\;\left(\%\right)=\frac{A_{ctr}-A_{samp}}{A_{ctr}}\times 100$$

where A_ctr_ is the absorbance of the negative control and A_samp_ is the absorbance of the sample. All the analyses were performed in triplicate.

#### 2.7.4 Cell metabolic activity evaluation

Cytocompatibility was evaluated on nucleus pulposus cells isolated and expanded as reported previously (Bari et al., 2018). Cells were seeded in a 96-well plate (10,000 cells/cm^2^) and cultured in Dulbecco’s Modified Eagle’s Medium High Glucose (DMEM-HG) with 10% v/v fetal bovine serum (FBS), 100 U/mL penicillin, 100 µg/mL streptomycin, 0.25 µg/mL amphotericin, 4 mM glutamine, 1 mM sodium pyruvate). Then cells were treated with 100 µL of samples solubilized in a culture medium (not supplemented with FBS) at the final concentrations of 0.8, 0.4, 0.2 and 0.1 mg/mL. Untreated cells were considered as control (100% of metabolic activity). After 24, 48 and 72 h of incubation, the supernatants were discarded, the cells were washed with PBS, and 100 μL of MTT solution (0.5 mg/mL) was added to each well. The MTT solution was removed after 3 h of incubation, and 100 μL of DMSO was added. The absorbance was measured by a microplate reader (Synergy HT, BioTek, Swindon, United Kingdom) at 570 nm and 670 nm (reference wavelength). The cell metabolic activity % was calculated using the following formula:

$$Cell\;metabolic\;activity\;\left(\%\right)=100\times \frac{A_{samp}}{A_{ctr}}$$

where A_sam_ is the absorbance of the tested samples, and A_ctr_ is the absorbance of the control. All experiments were performed in triplicate.

#### 2.7.5 Oxidative stress protection test

The ability to prevent oxidative stress damage was tested on nucleus pulposus cells. First, nucleus pulposus cells were seeded in a 96-well plate (5,000 cells/cm^2^) in a complete culture medium, as reported in Section 2.7.4. Then, cells were treated with 100 µL of culture medium (without FBS) containing samples at the final concentrations of 0.4 and 0.2 mg/mL. After 24 h, media were discarded, and cells were treated with H_2_O_2_ (1 mM). Cells not treated with samples and H_2_O_2_ were considered negative control (100% of the metabolic activity%), while cells not treated with samples but with H_2_O_2_ were considered positive control. Finally, an MTT test was performed after 24 h, and the cellular metabolic activity was calculated as reported in Section 2.7.4. All experiments were performed in triplicate.

### 2.8 Statistical analysis

The data with a normal distribution were analyzed with a linear generalized Analysis of Variance model (ANOVA), and the differences between the groups were evaluated using Fisher’s least significant difference (LSD) procedure. In detail, for each formulation, the data regarding the stability were elaborated considering the temperature and the time as fixed factors and the mean diameter, mode and d_50_ as response variables. For elaborating the antioxidant, anti-elastase and anti-tyrosinase data, the sample was considered a fixed factor, the concentration a covariate, and the activity (%) the response variable. Anti-elastase and anti-tyrosinase raw data were further elaborated with the Michaelis-Menten model kinetics (Chlapanidas et al., 2013) to calculate, for each concentration and sample, the K_m_ and V_max_ values. The K_m_ and V_max_ values were then analyzed with a linear generalized Analysis of Covariance model (ANCOVA), considering the sample as a fixed factor and the sample concentration as a covariate. Regarding the raw data about cell metabolic activity, the sample concentration and time were considered fixed factors, the concentration as a covariate and the cell metabolic activity (%) as the response variable. Finally, for the oxidative stress data, the H_2_O_2_ concentrations (0 or 1 mM) were considered fixed factors. The analyses have been conducted with STATGRAPHICS XVII (Statpoint Technologies, Inc., Warrenton, VA, United States) and Graph-Pad Prism software version 8.0.1. The statistical significance was set at *p* < 0.05.

## 3 Results and discussion

### 3.1 Characterization of the nanoparticles

Sericin nanoparticles were prepared using crocetin as a cross-linker (Perteghella et al., 2021). A cross-linker is necessary to stabilize the nanoparticles by forming bonds with the polar amino acid residues of sericin, especially with serine, aspartic acid and glycine, which are highly abundant (Wang et al., 2018). The effective cross-linking of sericin was assessed by analyzing the supernatants to isolate the components of the formulation. After treatment with HCl and hot water, the residual sericin appears as a light yellow-coloured glassy solid which has been weighed. For all the formulations and batches, the percentage yield is high (above 90%), confirming the excellent cross-linking process of the nanoparticles.

**FIGURE 1:**
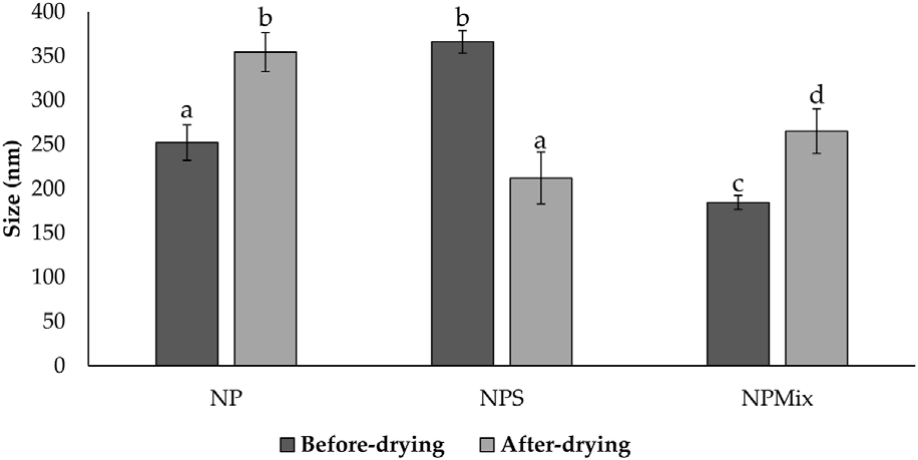
Size comparison of the nanoparticles of different batches of NP, NPS and NPMix before and after drying. Data are reported as mean value ± standard deviation, *n* = 9. Letters (a, b, c, and d) are used to compare the means of different groups. If the letters are different, there is a statistically significant difference between the means (*p* < 0.001); if the letters are the same, there is no statistically significant difference between the means (*p* > 0.05).

Then, nanoparticles were isolated and dispersed in water, sonicated and analyzed at the Nanosizer before and after the spray drying. Figure 1 compares the size of NP, NPS and NPMix. NP, before spray drying, has an average size of 252.03 ± 20.05 nm, significantly lower than NPS, where the dimensions before drying range from 366.1 ± 12.78 nm but higher than NPMix (184.53 ± 7.92 nm) (*p* < 0.05). The larger pre-drying sizes of NPS are probably due to the presence of the sericin in excess, which determines an aggregation of the dispersed nanoparticles. Following the spray drying process, for NP and NPMix, the dimensions of the nanoparticles significantly increased on average by about 100 nm (Figure 1). In detail, after drying, the NP and NPMix showed diameters of 354.53 ± 21.97 nm and 265.05 ± 25.31 nm, respectively. The largest diameter after spray drying could be due to a partial aggregation during the spray drying process. Conversely, for NPS, containing an excess of sericin, particle size has significantly reduced (from 366.1 ± 12.78 nm to 212.09 ± 29.20 nm, *p* < 0.05). This may be due to the shrinkage and collapse of sericin (in excess) following dehydration (Genç et al., 2009). NP and NPS are polydisperse before and after spray drying, as indicated by the PI value greater than 0.6. On the other hand, NPMix showed a PI value close to 1, which could be related to the possible presence in the dispersion of insoluble substances (like free crocetin) produced during the nanoparticle preparation.

**FIGURE 2:**
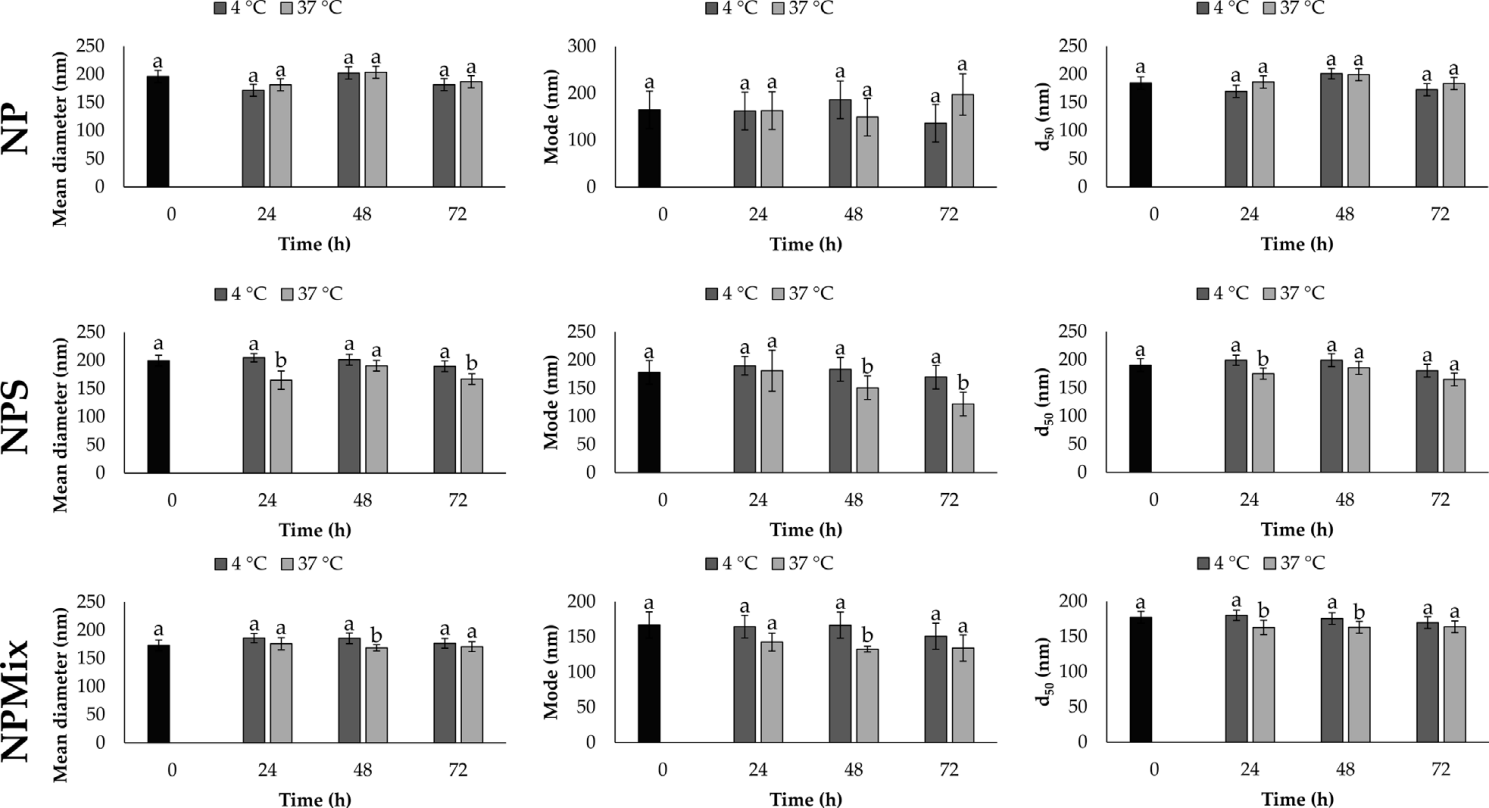
Mean diameter, mode and d_50_ for NP, NPS and NPMix formulations stored at four or 37°C for 24, 48 and 72 h. Data are reported as mean value ± LSD, Multifactor ANOVA, *n* = 15 for each formulation. Letters (a and b) are used to compare the means of different groups. If the letters are different, there is a statistically significant difference between the means (*p* < 0.05); if the letters are the same, there is no statistically significant difference between the means (*p* > 0.05).

The stability of each formulation was evaluated in a complete culture medium as a function of time (24, 48 and 72 h) and temperature (4°C and 37°C). Overall, the time and temperature significantly affect the size of the nanoparticles, which is different depending on the formulation (Figure 2). For NP, the mean diameter, mode and d_50_ did not change significantly as time and temperature varied (*p* > 0.05). Instead, a partial reduction in size was observed for NPS and NPMix over time, especially when stored at 37°C. The size reduction is likely due to the partial solubilization of nanoparticles in the culture medium.

**FIGURE 3:**
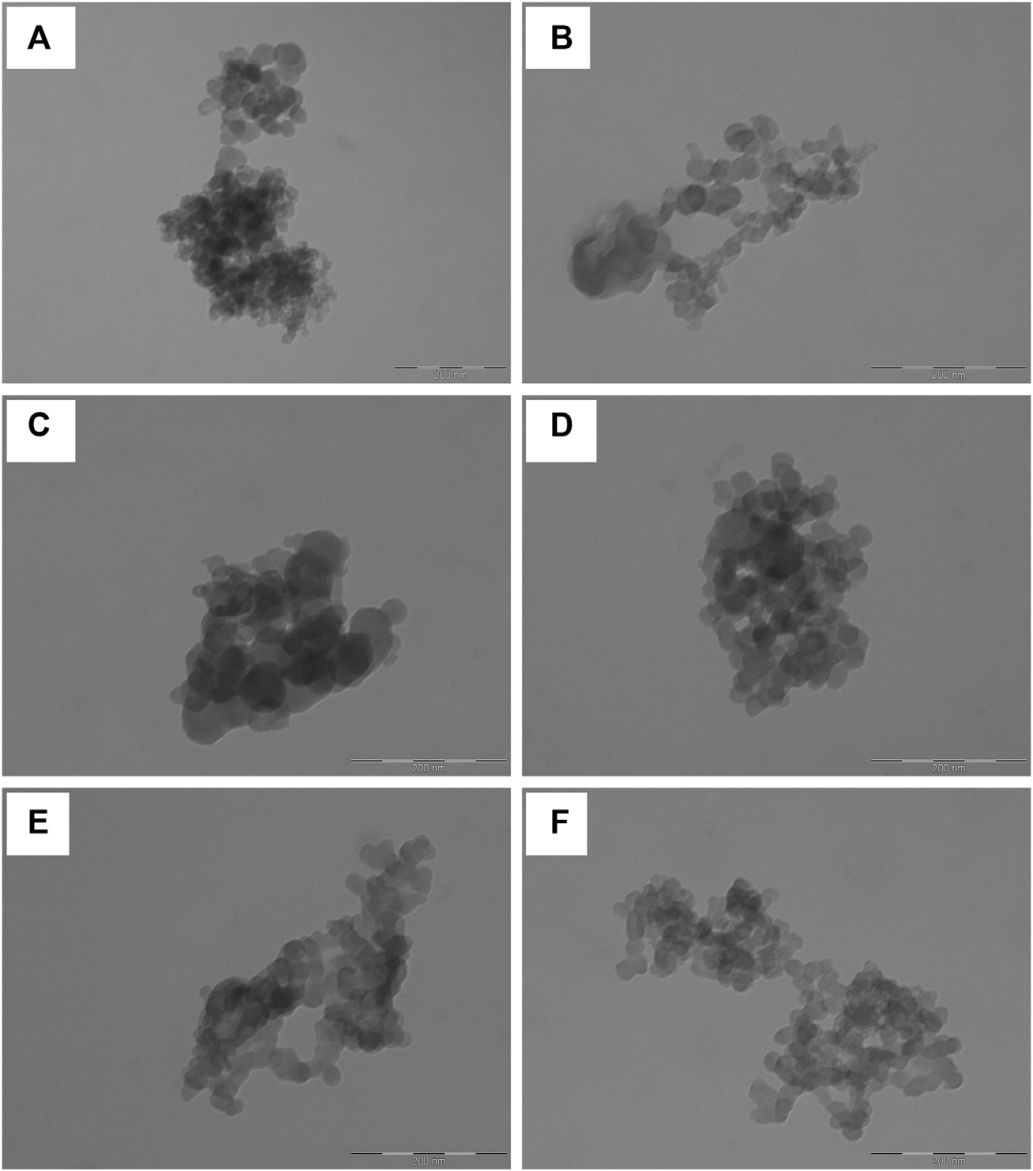
Representative TEM images of sericin nanoparticles obtained dispersing NP **(A and B)**, NPS **(C and D)** and NPMix **(E and F)** in deionized water. The scale bar is 200 nm.

At TEM, the nanoparticles appeared spherical and with a smooth surface; no pores or cavities were detected (Figure 3). Overall the appearance of NP was similar to the one reported by (Jovita et al., 2021), which used genipin as a cross-linker. Considering that the samples were prepared for TEM simply by dispersing the microparticle formulations in water, the nanoparticles appeared aggregated with sizes around 200 nm. The single nanoparticles were instead of about 50 nm. The bigger sizes detected during the particle size analysis and the stability study indicate that the nanoparticles in water or culture medium are solvated; thus, an extensive layer of water molecules adhere to their surface, forming polar interactions with sericin aminoacids.

NP showed a negative charge (−34.53 ± 0.84 mV) significantly higher than that of NPS and NPMix (−21.87 ± 0.35 and −24.20 ± 0.46 mV, respectively) (*p* < 0.05). The results of the nanoparticle characterization are summarised in Table 2.

**TABLE 2:** Average diameter and dimensional distribution (PI), before and after spray drying process, and zeta potential of the nanoparticles. Data are reported as mean values ± standard deviation, *n* = 3 or 9.

Formulation	Diameter (nm)			PI			Zeta potential (mV)
	Before	After		Before		After	
NP	252.03 ± 20.05		354.53 ± 21.97	1.03 ± 0.13	0.98 ± 0.19		−34.53 ± 0.84
NPS	366.10 ± 12.78		212.09 ± 29.20	0.92 ± 0.16	0.73 ± 0.16		−21.87 ± 0.35
NPMix	184.53 ± 7.92		265.05 ± 25.31	0.57 ± 0.09	0.59 ± 0.01		−24.20 ± 0.46

### 3.2 Characterization of the microparticles

During the spray drying process, the nanoparticles aggregate to form microparticles. The drying yield was calculated and reported in Table 3; the different composition of the feed suspension has led to a powder with different aerodynamics, which has determined a different deposition in the instrument and adhesive properties with the consequent impossibility of picking up all the powder. This has affected the production yield, which is, however, still good. This statement was confirmed by the higher yield obtained by nebulizing larger volumes of suspension (data not reported). Moreover, the different composition of the feed suspension affects the size of resulting microparticles: NP and NPS had similar size and size distribution (Table 3) with dvs around 2.30 µm (*p* > 0.05), whereas NPMix had the lowest dvs (*p* < 0.05).

**TABLE 3:** Yield of the spray drying process and size of microparticles, expressed as mean volume-surface diameter (dvs) and mode. Data are reported as mean values ± standard deviation, *n* = 3 or 9.

Formulation	Yield (%)	dvs (µm)	Mode (µm)	Crocin (% w/w)	Crocetin (% w/w)
NP	48.39 ± 7.99	2.24 ± 0.09	4.02 ± 0.40	-	-
NPS	51.76 ± 1.46	2.47 ± 0.11	4.40 ± 0.19	-	-
NPMix	72.50 ± 3.64	1.68 ± 0.04	2.63 ± 0.04	5.4 ± 1.9	12.9 ± 1.7

**FIGURE 4:**
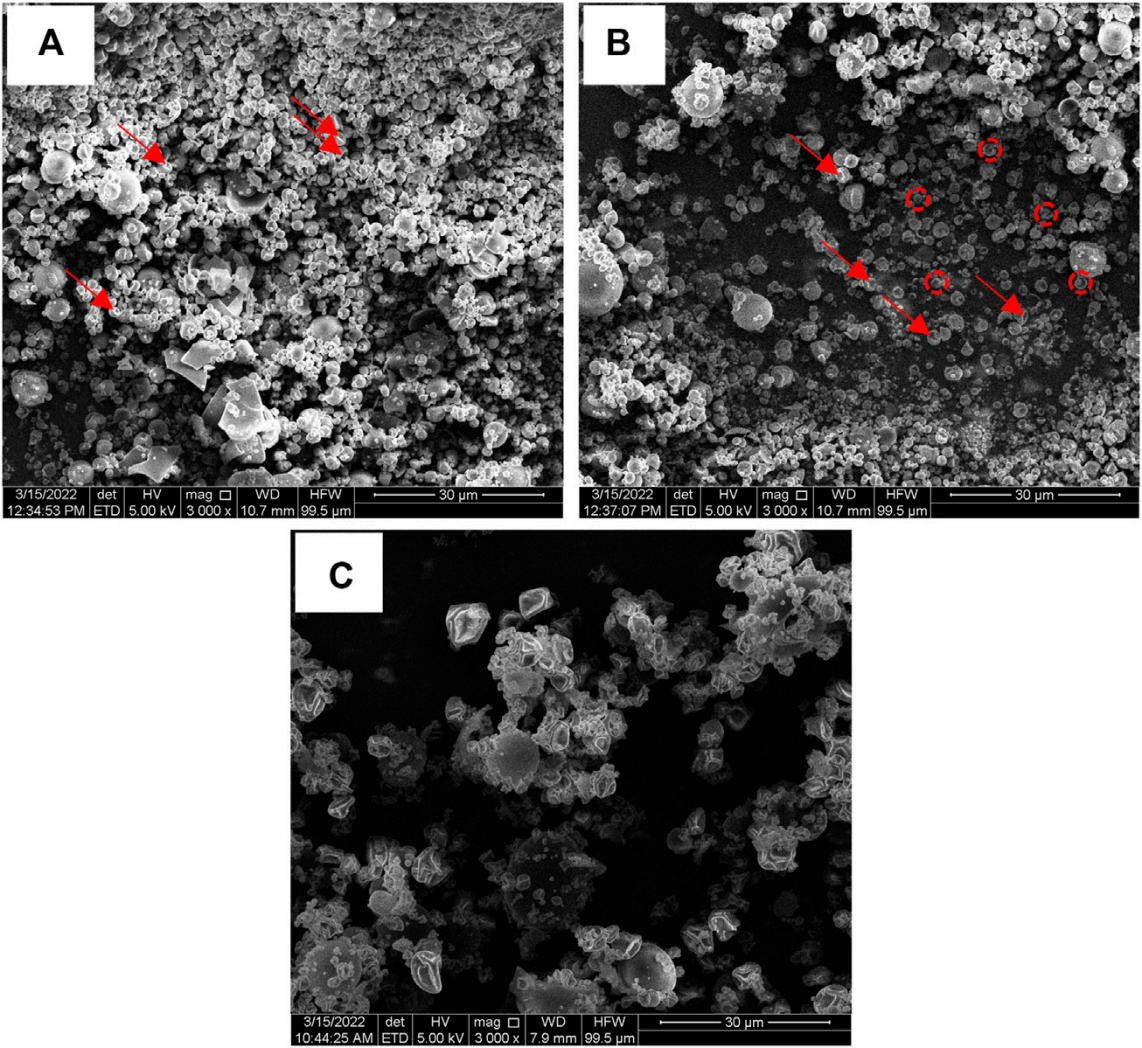
SEM images of NP **(A)**, NPS **(B)** and NPMix **(C)** formulations. 3000 × magnification. Red arrows point out some invaginations, while red circles detail particle roughness.

The morphology of microparticles in NP, NPS and NPMix formulations has been analyzed by SEM and reported in Figure 4. NP showed different populations of microparticles characterized by an almost spherical shape and smooth surface with invaginations (Figure 4A). Even for NPS, different microparticles were aggregated, some with a spherical shape and others with numerous invaginations and roughness (Figure 4B). On the other hand, the particles of the NPMix show a more homogeneous population with spherical dimensions, lamellar residues and a few microspheres with concavity (Figure 4C). This can be rationalized by considering the percentage of crocin and crocetin present in the reaction mix, which has been dried entirely without proceeding by separating the nanoparticles.

NPMix microparticles were also studied to evaluate free crocin and crocetin residue within the mix. Crocin and crocetin were separated from the NPMix according to their different solubility, which is linked to the pH of the extraction solvent: a slightly acidified aqueous solution was employed for the selective extraction of free crocin while the free crocetin fraction was isolated with a further extraction with NaOH aqueous solution. Although some HPLC/LC-MS-based methods have been recently reported in the literature for the quantification of crocins (Garcia-Rodriguez et al., 2014; Moras et al., 2018), taking into account the well-known stability concern of crocins (Reddy et al., 2020), a UV-Vis method for the quantification of total crocins and total crocetins was carried out (ISO, 1993). The measurement results are reported in Table 3 and show that a slight amount of both starting crocin and the linker crocetin is contained in the mixture of the samples.

### 3.3 *In Vitro* biological activity

*In vitro* biological activity regarding antioxidant, anti-elastase, and anti-tyrosinase activity has been tested. The batches of each formulation did not show significant differences in biological activity; therefore, the data are aggregated per formulation.

**FIGURE 5:**
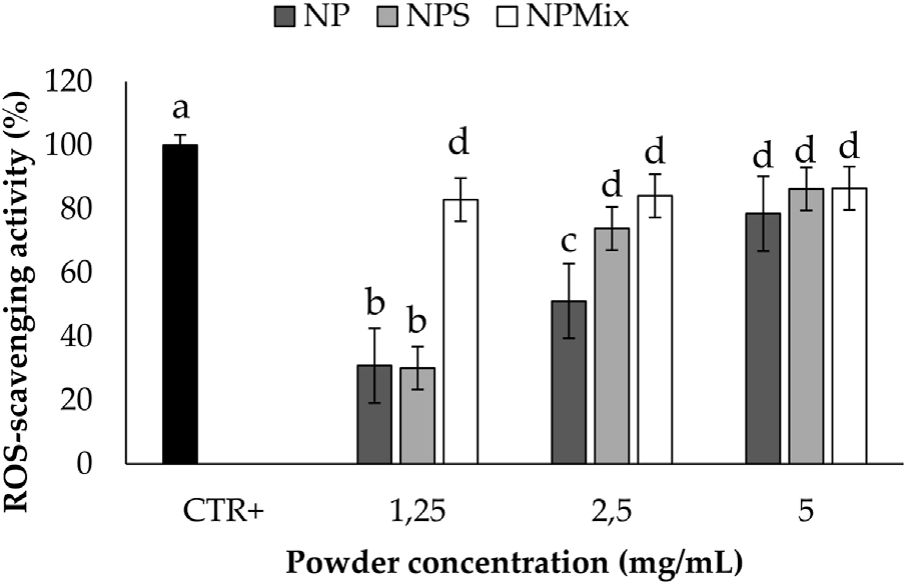
Results of the antioxidant activity of the samples. The positive control is ascorbic acid tested at 1.25 mg/mL in deionized water. Data are reported as mean value ± LSD, Multifactor ANOVA, *n* = 3. Letters (a, b, c, and d) are used to compare the means of different groups. If the letters are different, there is a statistically significant difference between the means (*p* < 0.001); if the letters are the same, there is no statistically significant difference between the means (*p* > 0.05).

The results of the antioxidant activity are shown in Figure 5. Some samples showed significantly different antioxidant properties depending on the concentration (*p* < 0.001). In detail, NP and NPS showed a dose-dependent antioxidant activity, while for NPMix, the activity reached its maximum even at the lowest concentration tested. This is likely because NPMix has an excess of crocetin, with high antioxidant properties even at the lowest concentration. In any case, the antioxidant activity of the samples was due to the simultaneous presence of sericin and crocetin. Indeed, the antioxidant activity of sericin is mainly due to the presence of residues of flavonoids but also to its amino acid sequence, which eliminates free radicals and ROS (Chlapanidas et al., 2013). As crocetin is a carotenoid, its antioxidant action results from its double carbon-carbon bonds interacting with each other *via* conjugation, causing electrons in the molecule to move freely across these areas of the molecule (Merhan, 2016).

**FIGURE 6:**
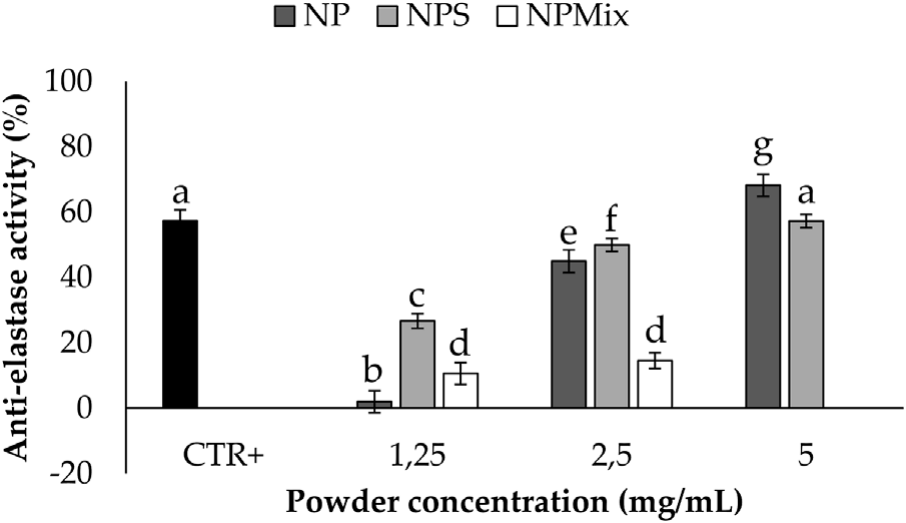
Results of the anti-elastase activity of the samples. The positive control is epigallocatechin gallate tested at a 7.2 mg/mL concentration in deionized water. Data are reported as mean value ± LSD, Multifactor ANOVA, *n* = 3. Letters (a, b, c, d, e, f and g) are used to compare the means of different groups. If the letters are different, there is a statistically significant difference between the means (*p* < 0.001); if the letters are the same, there is no statistically significant difference between the means (*p* > 0.05).

The anti-elastase activity of the samples is reported in Figure 6. A dose-dependent trend was observed for NP (*p* < 0.001), suggesting that sericin nanoparticles have a dose-dependent anti-elastase activity (NP = 100% w/w sericin nanoparticles). However, NPS showed the best anti-elastase properties, meaning that adding an excess of sericin during the formulation of microparticles increases the anti-elastase activity. Regarding NPMix, a low anti-elastase activity was observed at 1.25 and 2.5 mg/mL. As NPMix showed strong antioxidant properties, which are generally correlated with intense anti-elastase activity (Chlapanidas et al., 2013), it seems likely to suppose that the anti-elastase activity should increase by increasing the concentration tested. Unfortunately, this cannot be demonstrated because, at the highest concentration tested, the intense colour of crocetin masks the absorbance values (indeed, data are not reported in the graph). Therefore, it can be concluded that NPMix was the most active against ROS and the less promising in inhibiting the elastase enzyme.

K_m_ and V_max_ values were extrapolated using the Michaelis-Menten model kinetics to distinguish if the formulations inhibit or inactivate the elastase enzyme (Table 4). For NP, both K_m_ and V_max_ were significantly higher with respect to the negative control (enzyme without inactivator/inhibitors). Therefore, it can be concluded that NP, NPs and NPMix act as a general inactivator without competitive, non-competitive or mixed-type mechanisms, as these changes in K_m_ and V_max_ values are not typical for any of these mechanisms.

**TABLE 4:** K_m_ and V_max_ results for the enzyme elastase. Data are reported as mean values ± LSD. *p < 0.001 compared to CTR -.

Sample	K_m_	V_max_
CTR -	35.92 ± 53.76	0.884 ± 0.34
NP	163.45* ± 85.00	1.615* ± 0.55
NPS	16.07 ± 49.07	0.40 ± 0.31
NPMix	43.87 ± 53.76	0.97 ± 0.34

**FIGURE 7:**
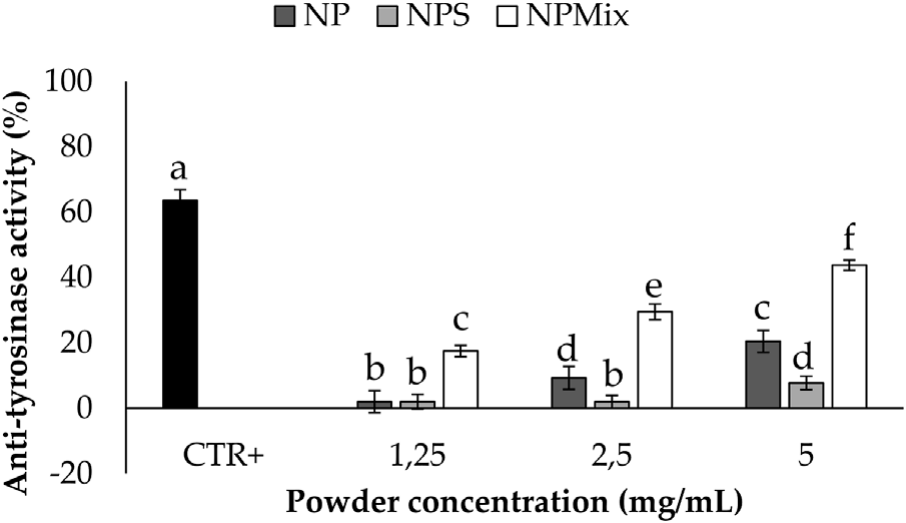
Results of the anti-tyrosinase activity of the samples. The positive control is arbutin tested at a 1.25 mg/mL concentration in deionized water. Data are reported as mean value ± LSD, Multifactor ANOVA, *n* = 3. Letters (a,b,c,d,e and f) are used to compare the means of different groups. If the letters are different, there is a statistically significant difference between the means (*p* < 0.001); if the letters are the same, there is no statistically significant difference between the means (*p* > 0.05).

The anti-tyrosinase activity of the samples is shown in Figure 7. A dose-dependent effect of the nanoparticles in the formulations was still observed (*p* < 0.001). In this case, crocetin was revealed to be the most active component in increasing the anti-tyrosinase activity, and as a result, NPMix, with the excess of crocetin, was the most active (*p* < 0.05).

Regarding K_m_ and V_max_ values, NPS significantly decreased K_m_ with respect to the control, while for NP, the V_max_ value increased significantly (Table 5). For NPMix, no significant differences were revealed from the negative control. Therefore, even in this case, any formulation showed competitive, non-competitive or mixed-type mechanism inhibitors against the enzyme tyrosinase.

**TABLE 5:** K_m_ and V_max_ results for the enzyme tyrosinase. Data are reported as mean values ± LSD. *p < 0.001 compared to CTR -.

Sample	K_m_	V_max_
CTR -	238.50 ± 111.11	4.45 ± 1.46
NP	365.13 ± 143.44	6.46* ± 1.89
NPS	92.21* ± 82.82	2.31 ± 1.09
NPMix	307.45 ± 101.43	3.98 ± 1.33

Overall, all the formulations showed antioxidant, anti-elastase and anti-tyrosinase activities, and NPMix was the most active, except in inhibiting the elastase enzyme. The anti-tyrosinase and anti-elastase activities may help prevent progressive structural failure during intervertebral disk degeneration. Indeed, elastase and matrix metalloproteinases are typically found within the extracellular matrix, and they can cleave elastin, collagen, fibronectin and other proteins. While physiologically, their action allows changes in shape, cell migration or tissue desorption required for tissue functionality, when overexpressed, they lead to tissue degeneration (Laronha and Caldeira, 2020). In addition, the formulations’ antioxidant properties may prevent oxidative stress’s effects on nucleus pulposus cells. Indeed, oxidative stress trigger apoptosis, directly or indirectly affecting the integrity of the cell membrane, disrupting sugar moiety or bases in DNA, causing proteolysis, and inducing lipid peroxidation (Nasto et al., 2013; Hou et al., 2014; Davalli et al., 2016; Ma et al., 2019).

**FIGURE 8:**
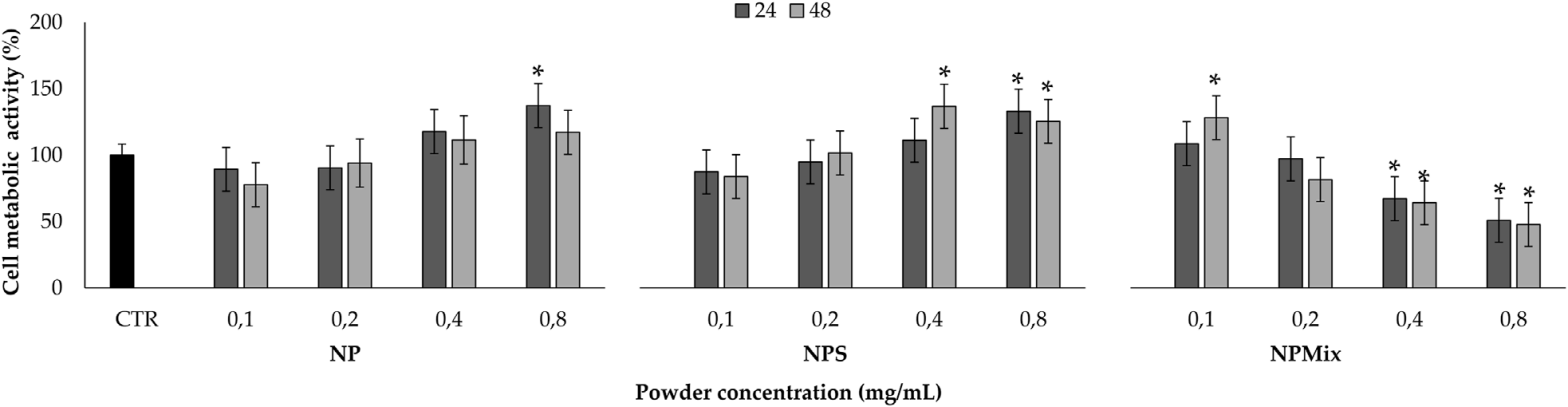
Sample cytocompatibility. Cells not treated with samples at 24 h were considered as controls (100% of the metabolic activity%). Data are reported as mean value ± LSD, Multifactor ANOVA, *n* = 3. *Indicates a significant difference with respect to the control (*p* < 0.05).

Before testing the formulations in protecting cells from oxidative stress damage, the cytocompatibility of the samples on human nucleus pulposus cells has been evaluated (Figure 8). The formulation and concentration significantly affected cell metabolic activity, while the treatment time effect was insignificant (*p* > 0.05). In detail, NP and NPS have no toxic effect in the concentration range tested, as the metabolic activity was above 80%. Also, by increasing the concentration of NPS (0.4 and 0.8 mg/mL), the cell metabolic activity was significantly stimulated compared to the control (*p* < 0.001). Conversely, NPMix has a cytotoxic effect, which depends on the concentration. Indeed, the metabolic activity was significantly lowered with respect to the control starting from 0.4 mg/mL, and the cytotoxic effect was observed at 0.8 mg/mL (*p* < 0.001). Therefore, the metabolic activity of cells treated with NP and NPS improves as the concentration increases, while for NPMix, as the concentration increases, toxic effects appear. Notably, the increased cell metabolic activity may suggest the ability of NP and NPS to sustain cell proliferation. For NPS, this effect is likely exerted by the excess of sericin, which is well known to act as a mitogen, stimulating cell mitosis (van der Valk et al., 2004; Terada et al., 2005). However, a synergistic effect with crocetin cannot be excluded as a similar effect was previously observed in another work from our group using sericin nanoparticles with other antioxidant natural compounds, like flavonoids, flavanol and tannins (Orlandi et al., 2020).

**FIGURE 9:**
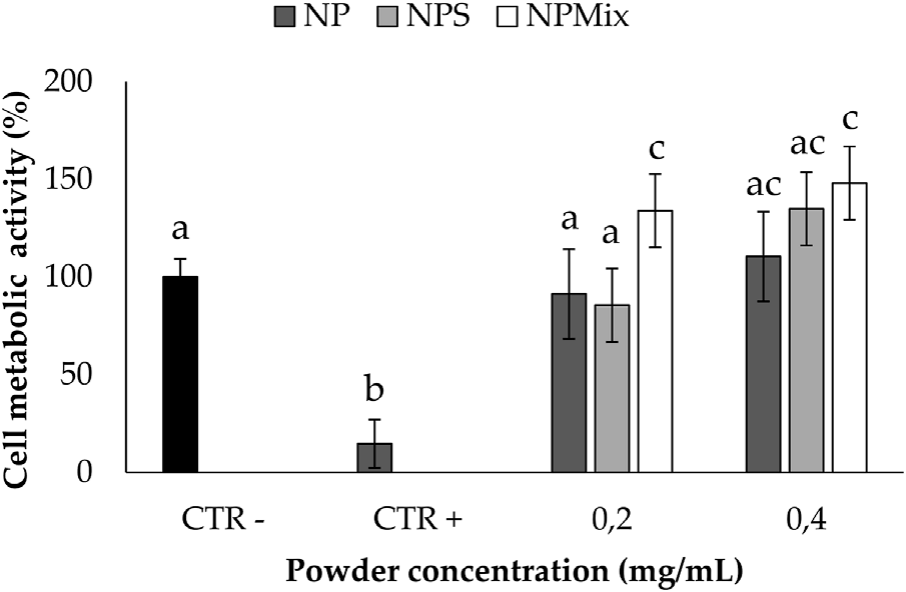
Oxidative stress protection test. Cells not treated with samples and H_2_O_2_ at 24 h were considered a negative control (100% of the metabolic activity%), while cells not treated with samples but with H_2_O_2_ were considered a positive control. Data are reported as mean value ± LSD, Multifactor ANOVA, *n* = 3. Letters (a, b, c, and ac) are used to compare the means of different groups. If the letters are different, there is a statistically significant difference between the means (*p* < 0.001); if the letters are the same, there is no statistically significant difference between the means (*p* > 0.05).

The ability to protect nucleus pulposus cells from oxidative stress damage is reported in Figure 9; only non-cytotoxic concentrations for all the samples were tested. Following the addition of H_2_O_2_ 1 mM, for the cells untreated with the formulations, the cell metabolic activity was significantly lowered (*p* < 0.05), thus confirming the suitability of the oxidative stress model. Conversely, all the samples could preserve the cell metabolic activity even after the treatment with H_2_O_2_, thus demonstrating the ability to prevent oxidative stress damage. Notably, the cell metabolic activity was significantly improved above 100% when using NPS at 0.2 mg/mL and NPMix at 0.2 and 0.4 mg/mL. Therefore, NPMix was the best in protecting cells from oxidative stress at both concentrations tested.

## 4 Conclusion

In this work, sericin and crocetin were combined to prepare “active per sé” nano-in micro formulations: at first, nanoparticles were prepared using crocetin as a cross-linker; then, microparticles were prepared by spray drying the only nanoparticles (NP) and the nanoparticles with an excess of sericin (NPS) or crocetin (NPMix). During the spray drying process, the small nanoparticles (about 250 nm) aggregated to form microparticles with a mean diameter of about 2 μm. The nanoparticles appeared spherical with a smooth surface, while microparticles were almost spherical, and their surface showed invaginations; lamellar residues and a few microspheres with concavity were observed for NPMix. Combining sericin and crocetin allowed for obtaining formulations with antioxidant, anti-elastase, and anti-tyrosinase activities. Furthermore, all the formulations effectively protected nucleus pulposus cells from the damage caused by oxidative stress. Overall, such results justify future investigations of these “active per sé” formulations in treating or preventing intervertebral disk degeneration and, eventually, deliver growth factors that can support cell viability and further promote tissue regeneration.

## Data Availability

The original contributions presented in the study are included in the article/supplementary material, further inquiries can be directed to the corresponding author.
